# Attenuation of inflammatory-mediated neurotoxicity by *Saururus chinensis* extract in LPS-induced BV-2 microglia cells via regulation of NF-κB signaling and anti-oxidant properties

**DOI:** 10.1186/1472-6882-14-502

**Published:** 2014-12-16

**Authors:** Byung-Wook Kim, Sushruta Koppula, Shin-Young Park, Jin-Woo Hwang, Pyo-Jam Park, Ji-Hong Lim, Dong-Kug Choi

**Affiliations:** Department of Biotechnology, Konkuk University, Chungju, Korea; Department of Biomedical chemistry, Konkuk University, Chungju, Korea

**Keywords:** Microglia, *Saururus chinensis*, Quercetin, LPS, NF-κB, Neurodegenerative disease

## Abstract

**Background:**

A *Saururus chinensis* Baill (SC) has been used by Native Americans, early colonists and practitioners of Korean traditional medicine for treating several diseases including cancer, rheumatoid arthritis and edema. The objective of this study was to evaluate the effects of SC extract in lipopolysaccharide (LPS)-stimulated neuroinflammatory responses in BV-2 microglial cells.

**Methods:**

The effects of SC on the LPS–induced neuroinflammatory responses in BV-2 microglial cells were assessed by Western blotting, RT-PCR and immunofluorescence labeling techniques. DPPH and alkyl radical scavenging assay was performed to evaluate the anti-oxidant effects. Comparisons between groups were analyzed using one-way analysis of variance followed by Dunnett’s multiple comparisons test using GraphPad Prism V5.01 software.

**Results:**

Pre-treatment with *SC* extract (1, 5 and 10 μg/mL) significantly (p < 0.001 at 10 μg/mL) and concentration dependently inhibited LPS-induced production of nitric oxide (NO), inducible NO synthase (iNOS), cyclooxygenase-2 (COX-2) and suppressed the inflammatory cytokine levels such as tumor necrosis factor-alpha and interleukin (IL)-6 in BV-2 microglial cells (p < 0.001 at 10 μg/mL). Further, SC suppressed the nuclear factor-kappa B (NF-κB) activation by blocking the degradation of IκB-α. SC also exhibited profound anti-oxidant effects by scavenging 1, 1-diphenyl-2-picrylhydrazyl (DPPH) (IC50: 0.055 mg/mL) and alkyl radicals (IC50: 0.349 mg/mL). High performance liquid chromatography finger printing analysis of SC revealed quercetin (QCT) as one of the major constituents compared with reference standard. QCT also inhibited the excessive release of NO, and inhibited the increased expressional levels of IL-6, iNOS and COX-2 in LPS-stimulated BV-2 cells.

**Conclusions:**

Our results indicated that SC inhibited the LPS-stimulated neuroinflammatory responses in BV-2 microglia via regulation of NF-κB signaling. The antioxidant active constituents of SC might be partly involved in delivering such effects. Based on the traditional claims and our present results SC can be potentially used in treating inflammatory-mediated neurodegenerative diseases.

## Background

Microglia, the immune cells of the central nervous system (CNS), plays an important role in neuroinflammation. It was well documented that microglial activation increases the risk for several CNS diseases such as Alzheimer’s disease, Parkinson’s disease (PD), multiple sclerosis, and Huntington’s disease [1–4]. Excessive activation of microglia due to pathogenic bacterial infection or injury releases proinflammatory mediators such as tumor necrosis factor (TNF)-α, interleukin (IL)-1β, IL-6, reactive oxygen species (ROS), nitric oxide (NO), inducible NO synthase (iNOS) and cyclooxygenase (COX)-2 [5]. Therefore, attenuation of neuroinflammatory events in microglia might be a promising strategy for preventing the progression of inflammatory-mediated neurodegenerative diseases.

*Saururus chinensis* Baill. (SC), a fragrant aquatic plant from the family Saururaceae has been used by Native Americans, early colonists and practitioners of Korean traditional medicine for treating a range of diseases including cancer, rheumatoid arthritis and nephritis-associated edema [6]. Pharmacological reports showed that SC possess anti-asthmatic [7, 8], anti-oxidative [9–11], anti-angiogenic [12], anti-inflammatory [7, 10, 12], anti-atopic [13], anti-cancer [14] and hepatoprotective properties [15]. Based on these reports, complementary studies are needed to determine whether the beneficial effects of SC are applicable to the treatment of neuroinflammatory and neurodegenerative diseases. In the present study, we investigated the anti-neuroinflammatory effects of SC extract against LPS-stimulated BV-2 microglial cells and explored the underlying mechanisms. The antioxidant status of SC was also evaluated using *in vitro* free radical scavenging assays. Further, to identify the major constituents in SC extract used in the study, high performance liquid chromatography (HPLC) fingerprinting analysis was performed.

## Methods

### Chemical materials

Lipopolysaccharide (LPS) (*E. coli* 0111:B4), Tween20, bovine serum albumin (BSA), dimethyl sulfoxide (DMSO), 3-[4,5-dimethylthiazol-2-yl]-2,5 diphenyl tetrazolium bromide (MTT) and quercetin (QCT) were purchased from Sigma-Aldrich (St. Louis, MO, USA). Six-well and 96-well tissue culture plates and 100 mm culture dishes were purchased from Nunc Inc. (North Aurora, IL, USA). DMEM containing 4.5 g/L D-glucose, L-arginine, 110 mg/L sodium pyruvate, and fetal bovine serum, as well as other cell culture reagents, TRIZOL and a Superscript™-III kit were obtained from Gibco/Invitrogen (Carlsbad, CA, USA). The 10× RIPA buffer was purchased from Millipore (Milford, MA, USA). The protease inhibitor cocktail tablets, phosphatase inhibitor cocktail tablets were supplied by Roche (Indianapolis, IN, USA). Antibodies to nuclear factor (NF)-κB p65 and COX-2 were obtained from Santa Cruz Biotechnology (Santa Cruz, CA, USA). Antibodies for iNOS, IκB-α, phosphor (p)-IκB-α, and β-actin were supplied by Cell Signaling Technology (Danvers, MA, USA).

### Preparation of the SC extract

Dried SC root material was purchased from a traditional herb market in Korea and authenticated by a taxonomist at the Plant Extract Bank, South Korea. A voucher specimen (CA02-047) was deposited at the institute’s herbarium, Konkuk University. To obtain the ethanol extract, 100 g of dried root material was added to 1 L of 95% ethanol, and the extraction was performed with sonication at room temperature for 15 min with an interval of 2 h using a 40 kHz model 8210R ultrasonic reactor (Branson Ultrasonic, Corp. Newtown, CT, USA). The procedure was repeated 15 times, and the mixture was filtered with 0.22 μM type GV film (Millipore, Milford, MA, USA). The filtrate was combined, and the final product was concentrated with a rotary evaporator, lyophilized, and stored at 4°C. The resulting powder (yield, 14.4 g) was re-dissolved in DMSO (0.1%) and filtered through a 0.22 μM filter before use. Standard QCT was purchased from Sigma-Aldrich for the comparative experimental studies. The SC extract and QCT were subjected to analytical HPLC (Waters, Sudbury, ONT, Canada), equipped with a UV detector. A GROM-SIL 120 C_18_ (4.0 mm × 250 mm) column was used.

### High-performance liquid chromatography (HPLC) finger print analysis

A Waters liquid chromatography system (Waters Associates Inc., Bedford, MA, USA), equipped with a double pump and a photodiode array detector was used. Separation was carried out on a Cosmosil 5C_18_-AR-II column (4.6 mm × 250 mm, 5 μm) with a column temperature set to 25°C. The mobile phases consisted of 0.4% aqueous phosphoric acid (A) and acetonitrile (B). For gradient elution the conditions were: 15% B (v/v) at 0–16 min, 15–25% B at 16–30 min, 25% B at 30–32 min, 25–30% B at 32–35 min, 30% B at 35–37 min, 30–33% B at 37–40 min, 33% B at 40–45 min, 33–48% B at 45–55 min, 48–55% B at 55–75 min, 55–80% B at 75–82 min, 80% B at 82–88 min, and the re-equilibration time of the gradient elution was 15 min. Flow rate was 0.8 mL/min, and injection volume was 10 μL. The detection wavelength for the QCT analyte was set to 360 nm. The absorption spectrum of the compound was recorded at 200–500 nm. The compound was identified by comparing the retention time and UV spectrum with those of standard markers.

### Cell culture and viability assay

BV-2 microglial cells were obtained and cultured as described previously [16]. Briefly, cells were seeded at a density of 5 × 10^5^ cells/mL, pretreated for 1 h with various concentrations of SC (1, 5, and 10 μg/mL) and then incubated for 24 h in medium containing LPS (100 ng/mL). Then, 0.5 mg/mL MTT was added to each well. After 4 h incubation at 37°C and 5% CO_2_, the supernatants were removed from each well, and the formed formazan crystals in the viable cells were dissolved in DMSO. Absorbance was determined at 540 nm using a microplate reader (Tecan Trading AG, Basel, Switzerland).

### NO production assay

NO production was assayed by measuring the levels of nitrite in culture medium using a colorimetric assay with Griess reagent [17]. BV-2 cells (5 × 10^4^ cells/mL) were seeded in 96-well plates in 100 μL culture medium and stimulated with LPS (100 ng/mL) for 24 h. The culture supernatant (50 μL) was reacted with an equal volume of Griess reagent (0.1% naphthylethylenediamine and 1% sulfanilamide in 5% H_3_PO_4_) in 96-well plates for 10 min at room temperature in the dark. Nitrite concentrations were determined using standard sodium nitrite solutions prepared in medium. The absorbance was determined at 540 nm using a microplate reader.

### Isolation of total RNA and reverse transcription polymerase chain reaction (RT-PCR)

Total RNA was extracted using TRIZOL reagent according to the manufacturer’s instructions. RNA (2.5 μg) was reverse-transcribed using a Superscript™-III kit according to the manufacturer’s instruction. PCR amplification was conducted using specific primers sets at annealing temperatures of 53.5–58°C for 20–30 cycles. The primer sequences used are presented in Table 1. The PCR conditions were described previously [18].

**Table 1:** **PCR primers used**

Gene target	Accession	Primer sequence	Size (bp)
**iNOS**	NM_010927	F 5′-CTTGCAAGTCCAAGTCTTGC-3	369
		R 5′-GTATGTGTCTGCAGATGTGCTG-3	
**COX**-**2**	NM_011198	F 5′-ACATCCCTGAGAACCTGCAGT-3′	414
		R 5′-CCAGGAGGATGGAGTTGTTGT-3′	
**TNF**-**α**	NM_01369	F 5′-TTCGAGTGACAAGCCTGTAGC-3	390
		R 5′-AGATTGACCTCAGCGCTGAGT-3′	
**IL**-**6**	NM_031168	F 5′-CATATGAGCTGAAAGCTCTCCA-3′	435
		R 5′-GACACAGATTCCATGGTGAAGTC-3′	
**GAPDH**	GU214026	F 5′-CCAGTATGACTCCACTCACG-3	378
		R 5′-CCTTCCACAATGCCAAAGTT-3	

### Western blot analysis

Cells were washed twice with PBS, placed at 4°C, and lysed for 10 min in lysis buffer (1× RIPA lysis buffer, protease inhibitor cocktail, phosphatase inhibitor cocktail). Lysates were centrifuged at 14000 rpm and 4°C, and the supernatants were collected for further analysis. Equal amounts of protein (40 μg) were separated electrophoretically by 10% sodium dodecyl sulfate-polyacrylamide electrophoresis, and the resolved proteins were transferred to polyvinylidene difluoride membranes (Millipore). The membranes were incubated for 1 h with 5% BSA in TPBT buffer to block nonspecific binding. The membranes were then incubated with primary antibodies to anti-iNOS, anti-IκB-α, anti-phospho-IκB-α (1:1000; Cell Signaling Technology), anti-β-actin (1:2000; Cell Signaling Technology) and anti-COX-2 (1:1000; Santa Cruz Biotechnology). The blots were visualized using PowerOpti-ECL (Animal Genetics Inc, Tallahassee, FL, USA) detection system according to the manufacturer’s procedure. Antibody-specific bands were scanned using a LAS-3000 luminescent analyzer, and quantified using Fuji Multigauge software V3.1 (Fuji, Tokyo, Japan).

### Double-immunofluorescence labeling assay

BV-2 microglia cells (1 × 10^5^ cells/well in a 12-well plate) were cultured on sterile cover slips in 12-well plates and treated with compounds and LPS to detect the intracellular location of the NF-κB p65 subunit. At 30 min after LPS treatment, the cells were fixed with methanol for 20 min at -20°C and washed three times with PBS. The fixed cells were then permeabilized with 1% Triton X-100 in PBS for 10 min at room temperature (RT), washed with 0.05% Tween20 in PBS for 5 min, followed by 0.05% Tween20/1% BSA in PBS for 5 min. The permeabilized cells were then treated with anti-NF-κB p65 (1:200; Santa Cruz Biotechnology) overnight at 4°C and washed with 0.05% Tween20/1% BSA in PBS for 5 min. The cells were then incubated in a 1:100 dilution of Alexa Fluor 568-labeled goat anti-mouse antibody (Invitrogen, Carlsbad, CA, USA) for 1 h at RT and washed with 0.05% Tween20 in PBS for 5 min, followed by PBS for 5 min. The cells were then stained with 1 μM Hoechst staining solution (Invitrogen) for 10 min at RT and then washed. Finally, all images were captured with a Carl Zeiss Axio 40 fluorescence microscope (Carl Zeiss, Oberkochen, Germany).

### Measurement of free radical scavenging activity

Free radical scavenging activity was evaluated using an electron spin resonance (ESR) spectrometer (JEOL, Tokyo, Japan). DPPH radical scavenging activity was measured using a method described previously [19]. A sample solution of SC (30 μL) was added to 60 μM DPPH (30 μL) in methanol and incubated for 2 min. Alkyl radicals were generated by 2,2′-azobis (2-amidinopropane) hydrochloride (AAPH). The reaction mixture containing 10 mM AAPH (20 μL), 10 mM 4-POBN (20 μL), and SC (20 μL) of various concentrations in PBS (20 μL, pH 7.4) was incubated at 37°C in a water bath for 30 min. The ESR spectrum was recorded for each radical using an ESR spectrometer.

### Statistical analysis

Data are expressed as means ± standard errors of mean (S.E.M). Each value was the result of three independent experiments (n = 3). Comparisons between groups were analyzed using one-way analysis of variance followed by Dunnett’s multiple comparisons test using GraphPad Prism V5.01 software (GraphPad Software Inc., San Diego, CA, USA). P-values < 0.05 were considered significant.

## Results

### SC extract attenuated LPS-induced nitrite production in BV-2 microglial cells

**Figure 1:**
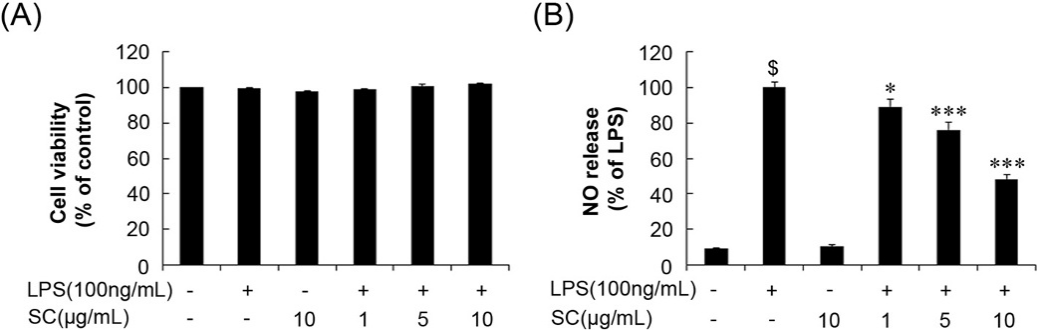
**Effect of the SC extract on nitrite production in LPS**-**stimulated BV**-**2 microglia.** Viability in SC-treated cells was evaluated using the MTT assay **(A)**. Cells were incubated with 1, 5, and 10 μg/mL of SC and LPS (100 ng/mL) for 24 h. Results are displayed as a percentage of control samples. Nitrite in the medium was determined by the Griess assay **(B)**. Results are displayed as a percentage of the LPS treated group. Data are mean ± standard error (n = 3) of three independent experiments. ^$^p < 0.001, compared with control group; *p < 0.05 and ***p < 0.001 compared with LPS-treated group. SC: *Saururus chinensis*, LPS: lipopolysaccharide, MTT: 3-[4,5-dimethylthiazol-2-yl]-2,5 diphenyl tetrazolium bromide.

To determine the cytotoxic potential of the SC extract, we evaluated its effects on the viability of BV-2 microglial cells at 5 × 10^4^ cells/mL. The MTT assay was performed 24 h after treatment with various concentrations (1–10 μg/mL) of SC in the presence or absence of LPS (100 ng/mL). Treatment with SC alone or with LPS (100 ng/mL) did not result in any signs of cytotoxicity at the indicated concentrations (Figure 1A). The vehicle (0.1% DMSO) used to dissolve SC also did not show any effect on overall cell viability as determined by the MTT assay (data not shown). To evaluate the effect of SC on NO production, BV-2 microglia cells were pretreated with 1, 5, and 10 μg/mL SC for 1 h and then stimulated with LPS (100 ng/mL). The culture medium from the cells was harvested and assayed for NO generation. Stimulation with LPS significantly increased (p < 0.001) the release of NO compared with that in the control group (Figure 1B). However, pretreatment with SC (1, 5, and 10 μg/mL) inhibited NO release in LPS-stimulated BV-2 cells in a concentration-dependent manner (Figure 1B).

### SC suppressed LPS-induced iNOS and COX-2 expression in BV-2 microglia cells

**Figure 2:**
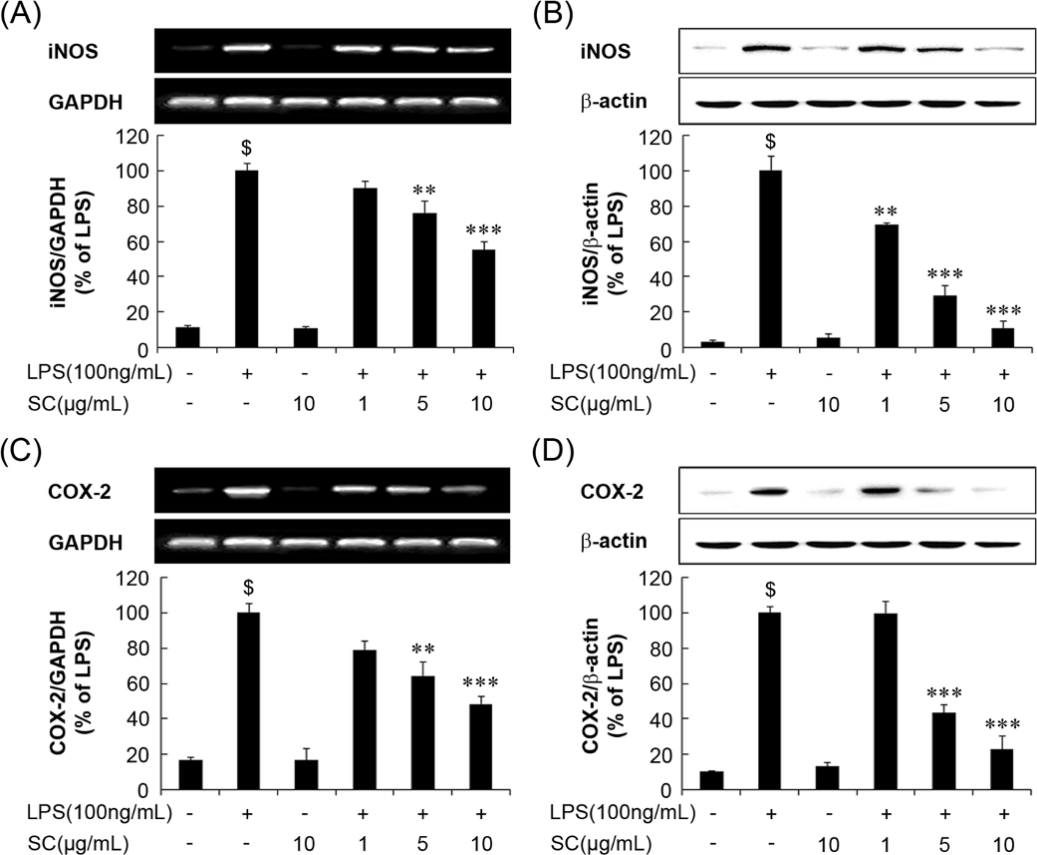
**Inhibition of inducible nitric oxide synthase** **(iNOS)** **and cyclooxygenase** **(COX)-** **2 mRNA and protein expression by the SC extract in LPS-** **stimulated BV**-**2 microglia. (A**, **C)** Cells were pretreated with the indicated concentrations of SC (1, 5, and 10 μg/ml) 1 h before stimulation for 6 h with 100 ng/mL LPS. Total RNA was prepared for reverse transcription-polymerase chain reaction analysis of iNOS and COX-2 gene expression from LPS-stimulated BV-2 microglia. **(B**, **D)** Cell lysates were electrophoresed, and iNOS and COX-2 expression was detected by a specific antibody. Data are mean ± standard error (n = 3) for three independent experiments. ^$^p < 0.001, compared with control group; **p < 0.01 and ***p < 0.001 compared with LPS-treated group. SC: *Saururus chinensis*, LPS: Lipopolysaccharide.

As shown in Figure 2A and Figure 2B, treatment with LPS increased iNOS mRNA expression at 6 h and iNOS protein expression at 18 h, respectively (p < 0.001). However, pretreatment with SC significantly attenuated LPS-induced iNOS mRNA and iNOS protein expression. These data indicate that SC suppresses LPS-induced NO production by inhibiting iNOS expression. COX-2 mRNA and protein levels were also significantly suppressed by SC in LPS-stimulated BV-2 cells. As shown in Figure 2C and Figure 2D, LPS treatment significantly up regulated COX-2 mRNA and protein expression. However, pretreatment with SC (1, 5, and 10 μg/mL) significantly (p < 0.01 at 5 μg/mL and p < 0.001 at 10 μg/mL) and concentration dependently reduced this increase in LPS-stimulated BV-2 cells. These data correlated well with the reduction in protein and corresponding mRNA levels.

### SC extract inhibited LPS-induced TNF-α and IL-6 production in BV-2 microglial cells

**Figure 3:**
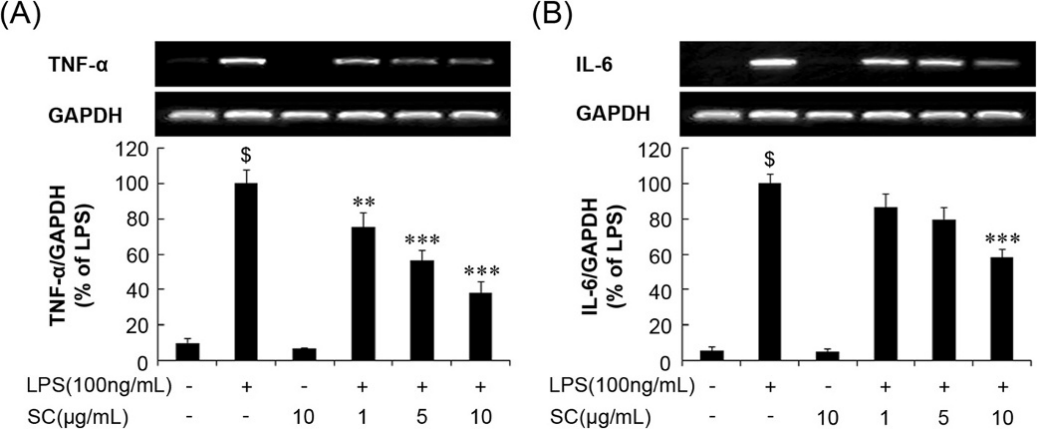
**Effect of the SC extract on pro-** **inflammatory cytokines in LPS**-**stimulated BV-** **2 microglia.** Cells were pretreated with the indicated concentrations of SC for 1 h before LPS (100 ng/mL) treatment. After incubation with LPS for 6 h, tumor necrosis factor (TNF)-α **(A)** and interleukin (IL)-6 **(B)** mRNA levels were determined by reverse transcription-polymerase chain reaction. Data are mean ± standard error (n = 3) for three independent experiments. ^$^p < 0.001, compared with control group; *p < 0.05 and ***p < 0.001 compared with LPS-treated group. SC: *Saururus chinensis*, LPS: lipopolysaccharide.

Proinflammatory cytokines such as TNF-α, IL-1β, and IL-6 are stimulators and/or co-stimulators of iNOS gene expression and play major roles in inflammatory disease [20, 21]. To investigate whether the SC extract had any effect on the production of proinflammatory cytokines (TNF-α and IL-6), BV-2 microglia were stimulated with LPS (100 ng/mL) in the presence or absence of SC at the indicated concentrations (1, 5 and 10 μg/mL). RT-PCR analysis showed that the mRNA levels of these cytokines increased 6 h after LPS treatment (p < 0.001). Pretreatment with SC for 1 h attenuated the upregulation of TNF-α and IL-6 in a concentration-dependent manner (Figure 3A and Figure 3B). These data suggest that SC inhibits the production of TNF-α and IL-6 by mediating regulatory gene expression at the transcriptional level, which is involved in the microglia-mediated inflammatory process.

### SC regulated NF-κB activation in LPS-induced BV-2 microglial cells

**Figure 4:**
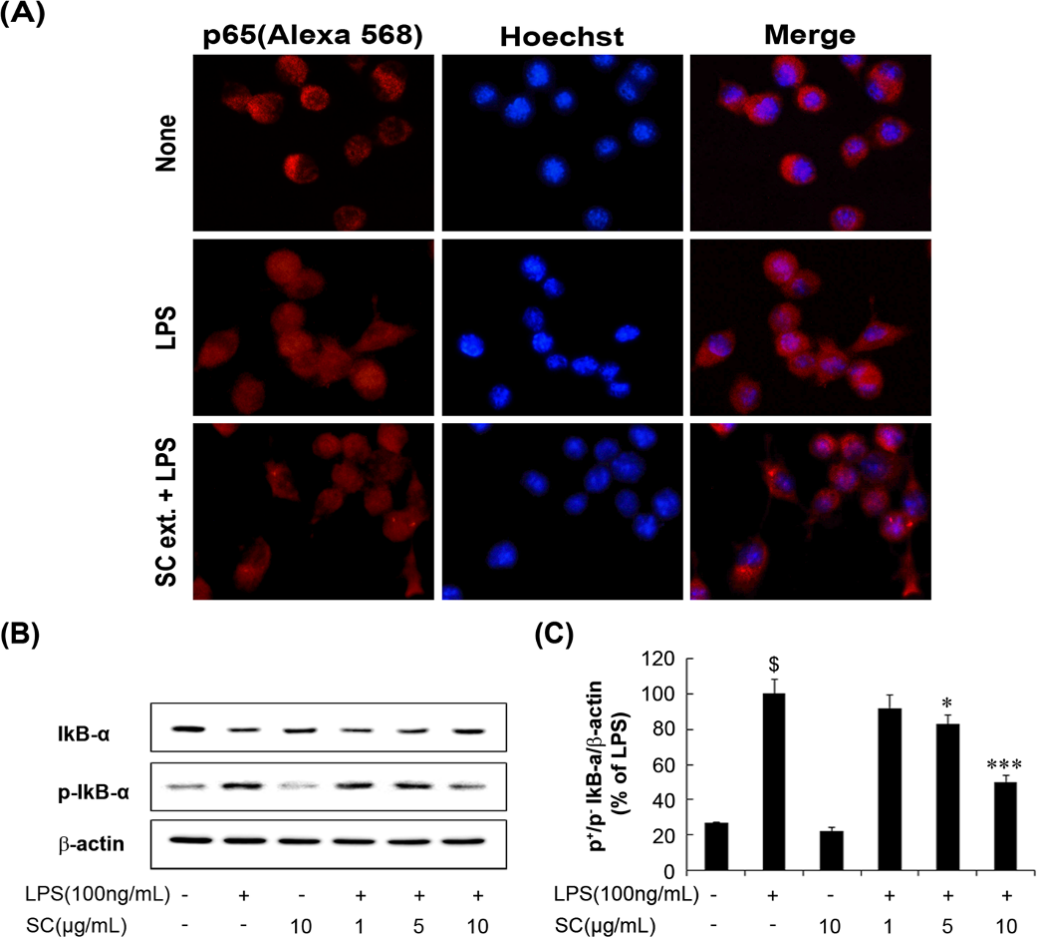
**Effect of the SC extract on nuclear factor** **(NF)-** **κB activity in LPS**-**stimulated BV-** **2 microglia.** BV-2 microglia cells were seeded at a density of 1 × 10^5^ cells/well on a 12-well plate. BV-2 microglia cells were stimulated with 100 ng/mL LPS in the absence or presence of the SC (10 μg/mL) extract that had been added 1 h before stimulation. At 30 min after LPS stimulation, sub-cellular location of the NF-κB p65 subunit was determined by double-immunofluorescence labeling assay **(A)**. Cells were treated with the indicated dose of SC 30 min before LPS (100 ng/mL) treatment. Total protein was subjected to 10% sodium dodecyl sulfate-polyacrylamide gel electrophoresis followed by Western blotting using anti-IκB-α and anti-p (phospho)-IκB-α **(B)**. Densitometric analysis of pIκB-α and IκB-α are represented in the panel. Results are expressed as a ratio of phospho/nonphospo IκB-α/β-actin **(C)**. Data are mean ± standard error (n = 3) of three independent experiments. ^$^p < 0.001, compared with control group; *p < 0.05 and ***p < 0.001 compared with LPS-treated group. SC: *Saururus chinensis*, LPS: lipopolysaccharide.

A previous study reported that LPS increases activation of the NF-κB subunit (via phosphorylation, ubiquitination, degradation and translocation of p65 and IκB-a) and regulates the expression of iNOS, COX-2, and other pro-inflammatory cytokines [22, 23]. Therefore, we performed double-immunofluorescence labeling assay studies to investigate whether the SC extract inhibited NF-κB translocation in LPS-stimulated BV-2 microglial cells. Interestingly, SC blocked nuclear translocation of the NF-κB p65 subunit (Figure 4A). To evaluate the effect of SC on IκB-α phosphorylation, BV-2 microglia were pretreated with SC (1, 5, and 10 μg/mL) for 1 h and then stimulated with LPS (100 ng/mL) for 30 min. IκB-α phosphorylation increased significantly (p < 0.001) after LPS treatment and translocation of the NF-κB p65 subunit was induced into the nucleus. SC pretreatment suppressed IκB-α phosphorylation in a concentration-dependent manner (Figure 4B and Figure 4C). These data demonstrate that SC inhibits NF-κB activation in LPS-stimulated BV-2 microglia and that this mechanism may contribute to the regulation of neuroinflammatory events.

### Free radical scavenging activities of SC

**Figure 5:**
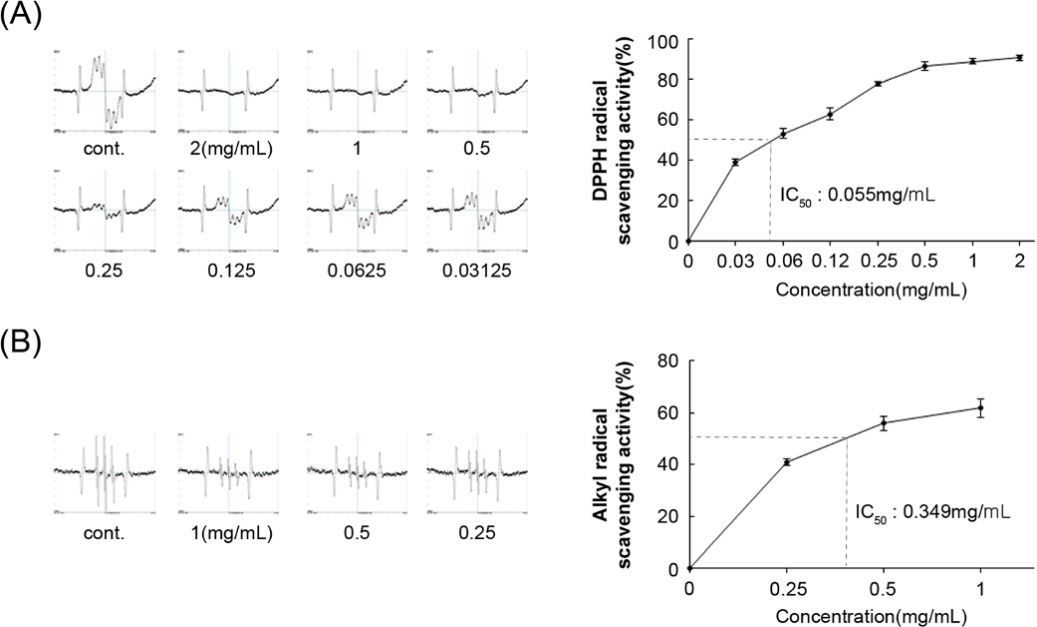
**Free radical scavenging effects of the SC extract. (A)** Left: Electron spin resonance (ESR) spectra of DPPH radical scavenging. Right: Relationship between the signal intensity of the DPPH radical and various concentrations of SC. **(B)** Left: ESR spectra of 2-(4-pyridyl-1-oxide)-N-t-butylnitrone (POBN)-trapped alkyl radical scavenging. Right: Relationship between the signal intensity of the POBN-alkyl radicals and the various concentrations of SC. SC: *Saururus chinensis*.

The potential of the SC extract to quench free radicals such as DPPH and alkyl radicals was investigated. DPPH is a stable free radical that accepts an electron or hydrogen radical to become a stable diamagnetic molecule. DPPH has been used to evaluate free radical scavenging activity of natural antioxidants. The capacity of SC to scavenge DPPH was measured by ESR spectrometry, and the results are shown in Figure 5A. DPPH radical scavenging activities of SC at various concentrations (0.031, 0.062, 0.125, 0.25, 0.5, 1, and 2 mg/mL) were 38.9 ± 1.5%, 53.2 ± 2.4%, 63.0 ± 3.1%, 77.8 ± 1.6%, 86.8 ± 2.2%, 88.9 ± 1.3%, and 90.8 ± 0.9%, respectively with an IC_50_ value of 0.055 ± 0.004 mg/mL. The alkyl radical spin adduct of 4-POBN/free radicals was generated from AAPH at 37°C for 30 min, and a decrease in ESR signals was observed with a dose increases of SC (Figure 5B). The alkyl radical scavenging activities of SC (025, 0.5, and 1 mg/mL) were 41.0 ± 1.3%, 56.0 ± 0.5%, and 62.1 ± 5.6%, respectively, with an IC_50_ value of 0.359 ± 0.07 mg/mL. These data show that SC possesses significant antioxidant activity.

### HPLC fingerprint analysis of SC and the effect of active constituent on proinflammatory cytokine expression in LPS stimulated BV-2 microglia

**Figure 6:**
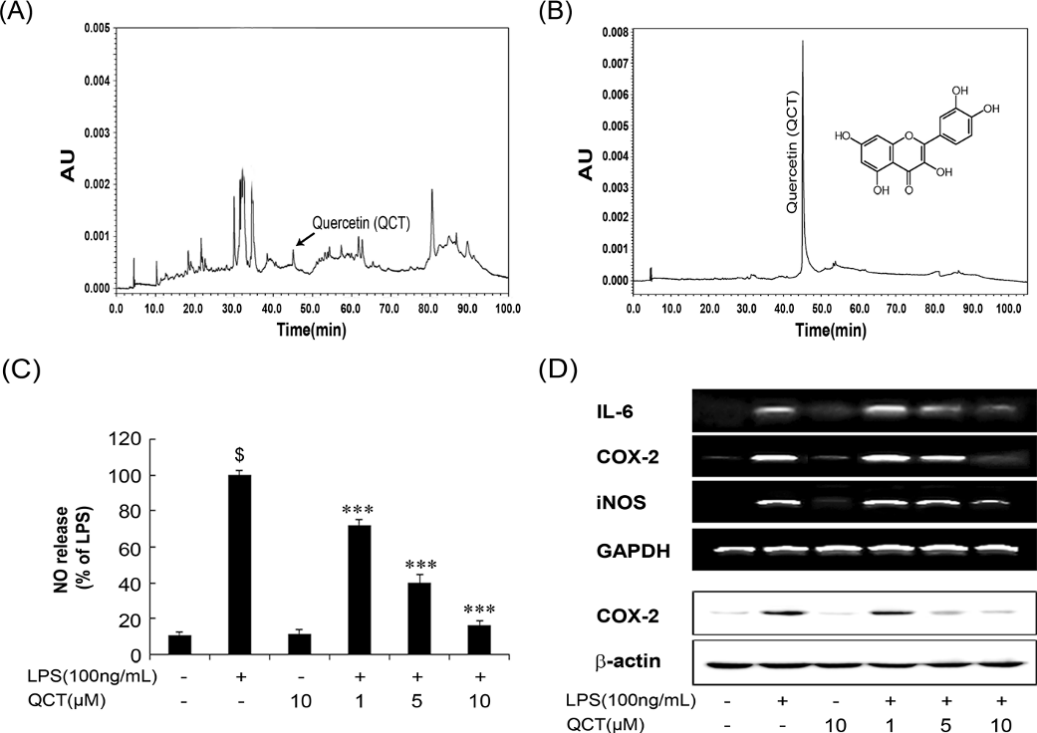
**High performance liquid chromatography** **(HPLC)** **fingerprinting of SC extract and the effect of QCT on nitric oxide** **(NO),** **interleukin** **(IL)-** **6,** **cyclooxygenase** **(COX)** **-2,** **and inducible nitric oxide synthase** **(iNOS)** **production in LPS-** **stimulated BV-** **2 microglia.** HPLC fingerprint analysis of SC **(A)** and QCT **(B)**. BV-2 cells were incubated for 24 h with LPS (100 ng/mL) in the presence or absence of the indicated concentrations of SC. The nitrite in the medium was determined by NO assay **(C)**. Several biomarkers were analyzed followed by reverse transcription-polymerase chain reaction and Western blotting **(D)**. Data are mean ± standard error (n = 3) of three independent experiments. ^$^p < 0.001, compared with control group; ***p < 0.001 compared with LPS-treated group. SC: *Saururus chinensis*, LPS: lipopolysaccharide, QCT: quercetin.

HPLC fingerprinting analysis of SC extract showed several peaks indicating a wide mixture of compounds. Earlier works indicated that SC extract contained polyphenolic compounds such as saucerneol D/F, sauchinone, manassantin A and B dineolignans and quercetin (QCT) flavonol glycosides [24–28]. Although the peaks obtained were below the level of quantification (Figure 6A), we compared with QCT as reference standard. Based on the retention time and UV spectrum we identified that SC extract used in our study contained QCT as one of the major constituents (Figure 6B). Further, to confirm the protective effects of QCT on NO release and cytokine expression, indicated concentrations of QCT (1, 5, and 10 μM) were pretreated to BV-2 cells stimulated with or without LPS (100 ng/mL). QCT alone at 10 μM did not exhibit any signs of toxicity in BV-2 cells (data not shown), however QCT (1, 5 and 10 μM) significantly and concentration dependently, suppressed LPS-induced production of NO (Figure 6C) and also attenuated the increase in IL-6, COX-2 and iNOS expression in a concentration-dependent manner (Figure 6D).

## Discussion

In the present study, we demonstrated that SC extract pre-treatment regulated the neuroinflammatory events in LPS-stimulated BV-2 microglial cells in several aspects. SC extract reduced LPS-stimulated NO production in BV-2 microglia cells in a concentration-dependent manner. SC also suppressed iNOS gene expression at the mRNA and protein levels. These results suggest that a significant decrease in NO release by SC extract is linked with inhibiting upstream iNOS gene expression.

COX is a key rate-limiting enzyme in the conversion of arachidonic acid to prostaglandins, which are lipid metabolites involved in several physiological and pathological processes, including neuroinflammation [29, 30]. COX-2 is mainly induced in response to inflammatory stimuli, which led to the concept that inhibiting COX-2 can reduce inflammatory processes in neurodegenerative diseases [31]. Several *in vivo* and *in vitro* studies have demonstrated that COX-2 is markedly up regulated in rodent brain microglia and in BV-2 microglia after LPS treatment [32–34]. In the current study we determined whether SC was associated with COX expression in LPS-stimulated BV-2 microglia cells. As a result, COX-2 mRNA and protein expression was dose-dependently suppressed by SC treatment (1, 5 and 10 μg/mL). Several reports have indicated that iNOS and COX-2 are induced in various types of CNS diseases [35, 36]. iNOS and COX-2 are expressed in glial cells of the substantia nigra in post-mortem patients with PD [37]. Therefore, attenuating the induction of iNOS and COX-2 in activated microglia could inhibit neuroinflammation.

It was well documented that pro-inflammatory cytokines such as TNF-α, IL-1β, and IL-6 activate iNOS gene expression in rodent glial and muscle cells [36, 38, 39]. The likelihood of the involvement of SC in attenuating such factors is supported by our observations that pro-inflammatory mediators produced by LPS treatment such as TNF-α and IL-6 were suppressed by SC in BV-2 microglial cells at mRNA levels. This indicated that SC extract may be an effective anti-neuroinflammatory therapeutic agent.

NF-κB is a key transcription factor that is activated by several cellular signal transduction pathways associated with the regulation of cell survival and the expression of proinflammatory cytokines and enzymes such as iNOS, IL-6 and TNF-α [40, 41]. The molecular mechanisms of NF-κB activation have been well studied, and they involve activation of a cascade of cytoplasmic proteins and nuclear translocation of the NF-κB p65 subunit [42, 43]. We found that SC extract attenuated LPS-induced IκB-α degradation as well as nuclear translocation of p65 in BV-2 microglia, indicating that SC extract inhibits iNOS and TNF-α gene expression in microglia and may be involved in the inhibition of NF-κB activation as a possible mechanism.

Previous studies have demonstrated that the specific iNOS inhibitors reduced NO and act as a potent antioxidant by inhibiting ROS production in LPS-simulated microglia [44, 45]. Reports also showed that several polyphenolic compounds with immense antioxidant potential possess anti-neuroinflammatory properties [46–48]. In light of such reports, in our present study, we found that the SC significantly scavenged DPPH and alkyl free radicals exhibiting potent antioxidant actions. The antioxidant potential of SC might partly be responsible in inhibiting neuroinflammation in LPS-stimulated BV-2 microglial cells.

SC contains several compounds such as saucerneol D/F, sauchinone, manassantin A and B dineolignans and quercetin (QCT) flavonol glycosides [24–28]. These compounds were well reported to possess various biological activities including anti-inflammatory activities [11, 26, 49–52]. In our present study HPLC fingerprint analysis showed that SC extract contained QCT as one of the major compounds compared with reference standard.

QCT, a flavonoid polyphenolic compound found in many natural herbs showed protective effects in both *in vivo* and *in vitro* neurodegenerative models based on its antioxidant function [46, 53–56]. Furthermore, QCT inhibited gene expression of NO, iNOS, COX-2, and IL-6 in LPS-stimulated BV-2 cells. Therefore, QCT and other antioxidant polyphenolic compounds present in the SC extract might partly contribute and enhance the anti-neuroinflammatory effects in LPS-stimulated BV-2 cells.

## Conclusions

In summary, SC exerted anti-neuroinflammatory actions by inhibition of major neuroinflammatory mediators and cytokines in LPS-stimulated BV-2 microglial cells. Mechanistic study showed that SC might act by regulating NF-κB signaling pathways and anti-oxidant defense mechanisms. The antioxidant potential of SC might also be responsible for such beneficial effects. Based on the traditional claims and the present data SC can be potentially used in treating several inflammatory- mediated neurodegenerative diseases.

## Abbreviations

BSA: Bovine Serum Albumin
COX-2: Cyclooxygenase-2
DMEM: Dimethyl Sulfoxide
FBS: Fetal Bovine Serum
GAPDH: Glyceraldehydes-3-Phosphate Dehydrogenase
HPLC: High-performance liquid chromatography
IĸB-α: Inhibitory protein kappa B alpha
IL-1β: Interleukin-1beta
IL-6: Interleukin-6
iNOS: Inducible Nitric Oxide Synthase
LPS: Lipopolysaccharide
MTT: p-nitrophenyl phosphate, and 3-(4,5-dimethylthiazol-2-yl)-2,5-diphenyltetrazolium bromide
NF-ĸB: Nuclear factor kappa B
NO: Nitric Oxide
PBS: Phosphate-buffered saline
PD: Parkinson’s Disease
RT-PCR: Reverse transcription polymerase chain reaction
SDS: Sodium Dodecyl Sulfate
TNF-α: Tumor necrosis factor-α.
